# Shrub establishment favoured and grass dominance reduced in acid heath grassland systems cleared of invasive *Rhododendron ponticum*

**DOI:** 10.1038/s41598-019-38573-z

**Published:** 2019-02-19

**Authors:** Gruffydd Lloyd Jones, Max Tomlinson, Rhys Owen, John Scullion, Ana Winters, Tom Jenkins, John Ratcliffe, Dylan Gwynn-Jones

**Affiliations:** 1 IBERS, Aberystwyth University, Penglais Campus, Aberystwyth, Ceredigion SY23 3DA UK; 2 Snowdonia National Park Authority, National Park Office, Penrhyndeudraeth, Gwynedd, LL48 6LF UK; 3 Forest Research, Thoday Building, Deiniol Road, Bangor, Gwynedd, LL57 2UW UK; 4 Natural Resources Wales, Maes y Ffynnon, Penrhosgarnedd, Bangor, Gwynedd, LL57 4DE UK

## Abstract

*Rhododendron ponticum* L. is a damaging invasive alien species in Britain, favouring the moist, temperate climate, and the acidic soils of upland areas. It outshades other species and is thought to create a soil environment of low pH that may be higher in phytotoxic phenolic compounds. We investigated native vegetation restoration and *R. ponticum* regeneration post-clearance using heathland sites within Snowdonia National Park, Wales; one site had existing *R. ponticum* stands and three were restoring post-clearance. Each site also had an adjacent, uninvaded control for comparison. We assessed whether native vegetation restoration was influenced post-invasion by soil chemical properties, including pH and phytotoxic compounds, using *Lactuca sativa* L. (lettuce) bioassays supported by liquid chromatography-mass spectroscopy (LC-MS^n^). Cleared sites had higher shrub and bare ground cover, and lower grass and herbaceous species cover relative to adjacent uninvaded control sites; regenerating *R. ponticum* was also observed on all cleared sites. No phenolic compounds associated with *R. ponticum* were identified in any soil water leachates, and soil leachates from cleared sites had no inhibitory effect in *L. sativa* germination assays. We therefore conclude that reportedly phytotoxic compounds do not influence restoration post *R. ponticum* clearance. Soil pH however was lower beneath *R. ponticum* and on cleared sites, relative to adjacent uninvaded sites. The lower soil pH post-clearance may have favoured shrub species, which are typically tolerant of acidic soils. The higher shrub cover on cleared sites may have greater ecological value than unaffected grass dominated sites, particularly given the recent decline in such valuable heathland habitats. The presence of regenerating *R. ponticum* on all cleared sites however highlights the critical importance of monitoring and re-treating sites post initial clearance.

## Introduction

Invasive alien species are increasingly recognised as a major threat to biodiversity and human welfare, and incur major economic costs^1^. Plant invasions have many detrimental effects, including preventing natural woodland regeneration, hybridisation with rare native species and shifting ecosystem processes such as carbon and nutrient cycling^2–4^. As a result, plant invasions are a global problem and one of the main threats to the survival of rare species and to ecosystem stability^3^.

*Rhododendron ponticum* L., an evergreen shrub of the Ericaceae family, is an example of a particularly damaging invasive plant in Britain. Native to the Caucasus region, the Black Sea Coast and the Iberian Peninsula, it was introduced to the British Isles in 1763 and has since invaded native woodlands and conifer plantations, upland grassland, heaths and bogs^5,6^. Once established in these habitats, *R. ponticum* reduces local biodiversity. It forms a dense canopy, under which only a few plant species can survive due to the low light intensity^2^. This inhibits the successful establishment of most tree and ground flora seedlings, therefore preventing native habitat regeneration^2,7,8^. The result is vast areas of dense *R. ponticum* monoculture and an ageing tree canopy, as mature plants are not replaced^9^.

There are many factors driving the success of *R. ponticum* in Britain. Despite its native Mediterranean habitat, it thrives in the moist, temperate British climate which enhances its seedling recruitment^6,8,10^. This is especially true along the wetter western coast and upland areas of Britain, where it is also well suited to the acidic soils^5,6^. *R. ponticum* has a high seed output with one bush able to produce over a million small, wind dispersed seeds, allowing for rapid spread over large distances^2,6,11^. Another factor contributing to the invasiveness of *R. ponticum* is its unpalatability to herbivores; its foliage contains high concentrations of phenolic compounds and grayanotoxins which protect it from grazing, and as a result it has very few natural enemies in the British Isles giving it a competitive advantage over native species^2,11–13^.

The invasiveness of *R. ponticum* and its damaging effects on native habitats may also be due to how it alters soil conditions; species belonging to the *Rhododendron* genus are reported to decrease soil pH and alter nutrient cycling^14–16^. Additionally, *Rhododendron* spp. may release bioactive compounds into the soil from the canopy, from decomposing plant material and by root exudation^17^. Some of these compounds, for example catechin, are considered to have phytotoxic properties, inhibiting the germination and growth of native plants^13,18^. Rotherham and Read^7^ found that *Festuca ovina* L. (Sheep’s fescue) root elongation was inhibited when growing in soil which contained *R. ponticum*, as well as in soil which had recently contained *R. ponticum* for two months, potentially the result of bioactive compounds introduced to the soil. Growth and germination inhibition has also been observed in bioassays using species such as *Lactuca sativa* L. (lettuce) growing in *Rhododendron* spp. leaf, organic matter and litter leachates^19,20^.

Plant-soil interactions such as these are considered important drivers in the invasion of other species^21^. Many invasive species are reported to introduce inhibitory compounds to the soil which impair the growth of native species^21^, a phenomenon described as the “novel weapons hypothesis”^18^. Our understanding of the role of these compounds in plant invasions is limited however, with many conflicting reports of their relevance within natural systems^22–24^. Furthermore, few studies have investigated the long-term legacy effect of these compounds post-clearance^21^. Secondary compounds can also cause shifts in the soil microbial community, as well as in nutrient and carbon cycling^25–27^. The above interactions result in the formation of novel, less chemically and biologically diverse soil conditions beneath the invasive plant^25^.

Increasing our understanding of these plant-soil interactions is important, as any legacy effects of invasion on the underlying soil will in turn influence the success of efforts to restore invaded sites to native habitats^25^. In order to successfully restore cleared sites, native plants must be able to grow on the altered soil after *R. ponticum* removal. Most current restoration projects involve cutting *R. ponticum* and either burning or chipping the biomass^28,29^. Restoration is often slow however, as the seedbanks of invaded areas are often depleted of native species due to the duration of *R. ponticum* dominance^30–32^. Maclean *et al*.^31^ for example found that the understory vegetation of a Scottish Atlantic oak woodland had not fully recovered 30 years post-clearance.

*R. ponticum* regeneration on sites post-clearance is also common as its cut stumps readily re-sprout. These re-growing stems often require treatment post-clearance with herbicide, either by stem-injection or spraying onto the foliage^29^. Additionally, the bryophytes which first colonise the bare ground provide a suitable substrate for the germination of *R. ponticum* seeds arriving from adjacent uncleared land^6^. The need for continued monitoring and re-treating of sites post-clearance further increases the cost of managing *R. ponticum*. Therefore, finding ways of improving the efficiency and success of regeneration projects is imperative.

Our investigation studied the restoration of native vegetation and *R. ponticum* regeneration on cleared heathland sites within Snowdonia National Park, where it is particularly invasive. Percentage cover of native vegetation was measured on sites restoring post-clearance, and the extent of *R. ponticum* regeneration was also measured. Leading from this, the soil chemical properties of these sites were analysed, and *L. sativa* bioassays were conducted on soil leachates. These analyses aimed to determine whether the soil chemical legacy of *R. ponticum* influenced native vegetation restoration post-clearance, as the impact of inhibitory compounds on vegetation restoration post invasive plant clearance remains inconclusive.

It was hypothesised that the effect of *R. ponticum* invasion will remain post-clearance, increasing shrub cover and negatively affecting other plants. This was expected as the presence of *R. ponticum* over a prolonged period may create soil conditions favourable to ericaceous species and less favourable to other species. It was further hypothesised that reportedly phytotoxic phenolic compounds originating from *R. ponticum* will remain in the soil post-clearance, having a negative effect on the seed germination in *L. sativa* bioassays.

## Results

### Native vegetation restoration

**Figure 1:**
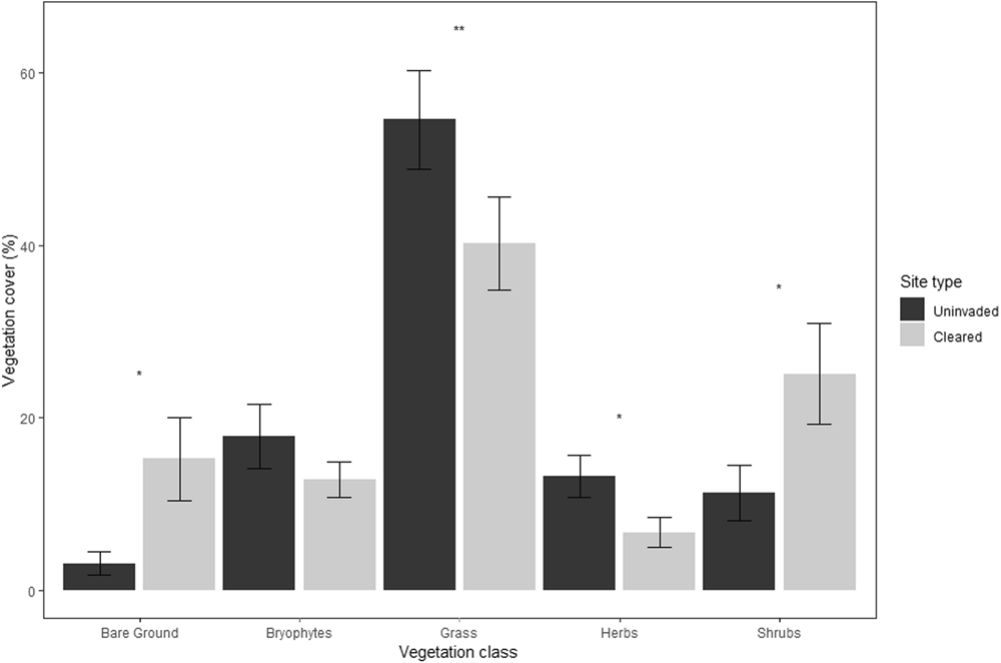
The mean percentage cover of the different vegetation classes on the cleared and uninvaded control sites. The error bars represent the SEM. Significance between site type is denoted by: *(P < 0.05) and **(P < 0.01) following analyses in mixed effects models, with site included as a random effect.

This study investigated how the cover of different native vegetation classes varied between sites cleared of *R. ponticum* and adjacent uninvaded control sites. Mixed effect models were fitted for each individual vegetation class, with site included as a random effect. These models revealed grass percentage cover was significantly lower (P = 0.035, n = 30) on the cleared sites relative to the uncleared control sites, whereas shrub cover (excluding *R. ponticum*) was significantly higher (P = 0.018, n = 30) (Fig. 1). Additionally, cleared sites had higher bare ground cover (P = 0.015, n = 30) and lower herb cover (P = 0.012, n = 30). No difference was observed in bryophyte cover (P = 0.167, n = 30). Vegetation cover for the uncleared site was not included in the analyses as the ground beneath the existing *R. ponticum* stands was almost entirely bare, with only a thick layer of leaf litter covering the soil.

**Figure 2:**
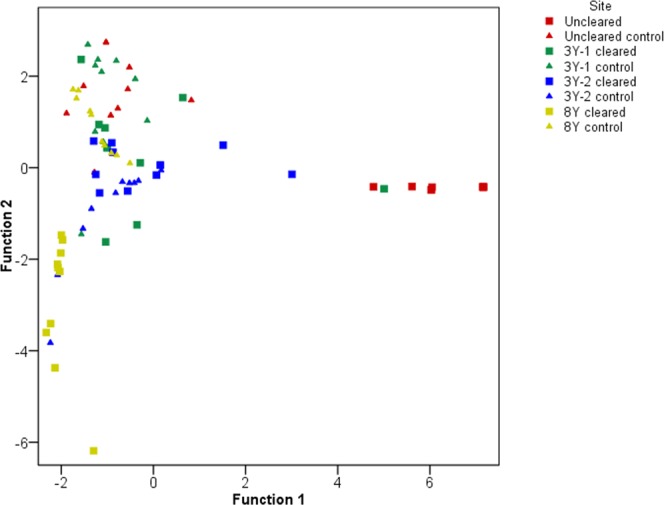
Plot of a canonical variate analysis comparing the vegetation classes of each site. The variables included were the relative cover of shrubs, bare ground, herbs, grasses and bryophytes. The percentage data were arcsine transformed prior to analysis. Different colours indicate different sampling sites, which included an uncleared site, two sites cleared three years prior to sampling (3Y-1 and 3Y-2) and one site cleared eight years prior to sampling (8Y). Square points denote the invaded and cleared sites, whereas triangles represent the adjacent uninvaded control sites. Wilks’ lambda test was used to test for significant differences between the means (P < 0.05).

Following on from the above, Wilks’ lambda test detected significant differences in percentage cover of vegetation classes between different sites (P < 0.001, n = 8), which form distinct clusters in a canonical variate analysis (CVA) (Fig. 2). The uncleared *R. ponticum* site was separated from its control and the other sites along function 1. The uninvaded control sites were closely grouped, suggesting the cover of vegetation classes prior to invasion were similar. In terms of the cleared sites, both 3Y-1 and 3Y-2 sites were similar, whilst the cleared 8Y site was separated from the other invaded sites, as well as from its adjacent control site, along function 2.

### *R. ponticum* regeneration on cleared sites

**Figure 3:**
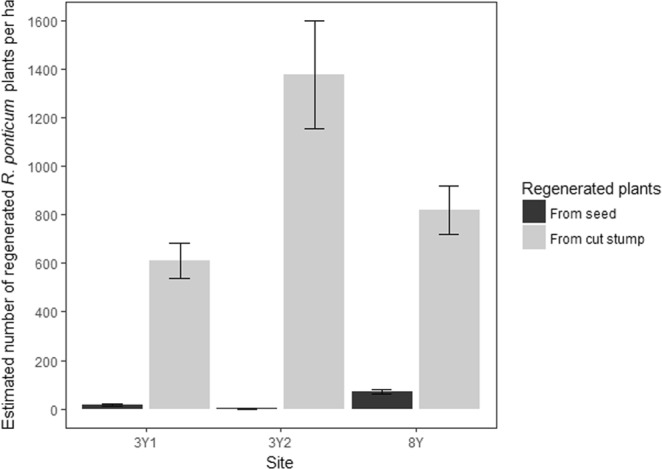
The estimated number of regenerated *R. ponticum* plants from seed and from cut stumps per hectare at each of the three cleared sites. Sites 3Y-1 and 3Y-2 were both cleared three years prior to sampling, with site 8Y cleared eight years prior to sampling. The error bars represent the SEM.

Regenerating *R. ponticum* was found at each of the three cleared sites (Fig. 3). Mixed model analysis, with site included as a random effect, revealed the majority (97%) of these regenerating plants were re-sprouting shoots from cut stumps, with only 3% originating from seed (P < 0.001, n = 18).

### Soil chemistry change in cleared sites

No water-soluble phenolic compounds detected in *R. ponticum* foliage were identified in any of the soil samples collected (Table 1). However, grayanotoxins were detected in soil samples from beneath existing stands at the uncleared site as well as the cleared parts of 3Y-1, 3Y-2 and 8Y. As no identifiable compounds were extracted from soil in water, methanolic extractions of uncleared soil were conducted to test whether any compounds were present in the soil. These extractions revealed six compounds found in *R. ponticum*, which were quinic acid, gallic acid and catechin, and three unidentified compounds also found in extracts of *R. ponticum* leaves (Table 1).

**Table 1:** A list of the compounds previously found in *R. ponticum* identified in the distilled water and methanolic extractions of the litter and soil samples.

Sample	No.	Compound name	t_r_	MW	Parent ion *(m/z)*	MS^2^ *m/z* (relative abundance)	λ_max_(nm)
					[M−H] [M + H]		
Litter water leachate	1	Quinic acid	1.2	192	191	173 (100), 147 (55), 111 (20), 127 (5)	264
	2	Caffeic acid derivative	11	432	431	179 (100), 225 (45), 161 (10), 143 (10)	277
	3	Grayanotoxin III	11.3	334	335	299 (100), 317 (40), 281 (30)	—
	4	Grayanotoxin II	19.2	334	335	299 (100), 317 (35), 281 (30)	—
	5	Unknown	32.6	266	265	97 (100), 265 (25), 266 (10)	275
	6	Unknown	32.8	294	293	97 (100), 293 (5)	269
	7	Unknown	33.7	408	407	407 (100), 327 (25)	309
Litter methanol extract	1	Quinic acid	1	192	191	147 (100), 111 (5), 147 (5), 127 (5), 85 (5)	264
	2	Gallic acid	2	170	169	125 (100), 169 (5)	275
	3	Gallic acid-*O*-hexoside	2.3	332	331	313 (100), 271 (90), 168 (70), 169 (50), 241 (23)	277
	4	3-*O*-Caffeoylquinic acid	5	354	353	191 (100), 179 (45)	282, 321
	5	(Epi)catechin-(4,8′)-(epi)catechin	5.9	578	577	425 (100), 407 (35), 451 (25), 289 (20), 559 (12)	276
	6	Methyl gallate	6.6	184	183	183 (100), 168 (55), 123 (45)	275
	7	Coumaroylquinic acid	6.7	338	337	163 (100), 191 (10)	283
	8	Catechin	6.9	290	289	289 (100)	279
	9	5-O-Caffeoylquinic acid	8	354	353	191 (100), 353 (10), 176 (5)	283, 321
	10	Caffeic acid derivative	11	432	431	179 (100), 225 (45), 161 (10), 143 (10)	277
	11	Grayanotoxin III	11.4	334	335	299 (100), 317 (40), 281 (30)	—
	12	Myricetin-*O*-hexoside	14.4	480	479	316 (100), 317 (85)	269
	13	Quercetin-*O*-galloyl-hexoside	15.2	616	615	463 (100), 464 (20), 301 (12)	270
	14	Myricetin-*O*-pentoside	15.8	464	463	316 (100), 317 (70)	301, 349
	15	Quercetin-*O*-hexoside	16.6	464	463	301 (100), 300 (25)	263, 351
	16	Quercetin-*O*-pentoside	18.4	434	433	301 (100), 300 (90), 302 (10)	255, 352
	17	Quercetin-*O*-rhamnoside	18.9	448	447	301 (100), 300 (25)	255, 349
	18	Grayanotoxin II	19.7	334	335	299 (100), 317 (35), 281 (30)	—
	19	Laricitrin-*O*-pentoside	20.2	464	463	331 (100), 316 (5)	271
	20	Unknown	32.6	266	265	97 (100), 265 (25), 266 (10)	275
	21	Unknown	32.8	294	293	97 (100), 293 (5)	269
	22	Unknown	33.7	408	407	407 (100), 327 (25)	309
Soil water leachate	1	Grayanotoxin III	11.3	334	335	299 (100), 317 (40), 281 (30)	—
	2	Grayanotoxin II	19.2	334	335	299 (100), 317 (35), 281 (30)	—
Soil methanol extract	1	Quinic acid	1	192	191	147 (100), 111 (5), 147 (5), 127 (5), 85 (5)	264
	2	Gallic acid	2	170	169	125 (100), 169 (5)	275
	3	Catechin	6.9	290	289	289 (100)	279
	4	Unknown	32.6	266	265	97 (100), 265 (25), 266 (10)	275
	5	Unknown	32.8	294	293	97 (100), 293 (5)	269
	6	Unknown	33.7	408	407	407 (100), 327 (25)	309

Also included are the compounds’ retention times (RT), molecular weights (MW), parent ion *m/z* and MS^2^ fragmentation data in either negative or positive ion mode.

Litter samples from the uncleared site contained a total of seven water-soluble compounds found in *R. ponticum*. These included quinic acid, a caffeic acid derivative, grayanotoxin-II, grayanotoxin-III and the same three unidentified compounds detected in the soil extracts. Methanolic extractions of these litter samples were also conducted, revealing a total of 22 compounds. These included glycosides of flavonoids such as quercetin, laricitrin and myricetin (Table 1).

Soil pH was significantly lower on the *R. ponticum* invaded and cleared sites relative to the adjacent uninvaded control sites (P < 0.001, n = 40) (Table 2). Sites were included as a random effect in the model used for this analysis.

**Table 2:** Mean soil pH of the invaded and control areas of each of the four sites.

	Uncleared	3Y-1	3Y-2	8Y
Treatment	3.9	4.1	4.1	3.8
Control	4.4	4.6	4.9	4.6

Prior to calculating means and statistical analysis, the data were log-transformed in R. Following this, the data were transformed back to the logarithmic scale of pH units for displaying. Soil pH of the uninvaded control sites was significantly higher than the uncleared and cleared sites (P > 0.05) following analysis in a linear mixed effect model, with site included as a random effect.

### Soil leachate bioassays

*L. sativa* bioassay germination data in soil leachates from cleared and uninvaded soils were analysed in a linear mixed effects model, with site included as a random effect. Germination of *L. sativa* was not suppressed when growing in soil leachates from the cleared sites relative to uninvaded control soil leachates (P = 0.857, n = 30) (Table 3).

**Table 3:** The mean germination (±SEM) of the *L*.

Site	Treatment	Mean germination (%)
3Y-1	Cleared *R. ponticum*	75 ± 4
	Uninvaded	83 ± 2
3Y-2	Cleared *R. ponticum*	76 ± 3
	Uninvaded	75 ± 3
8Y	Cleared *R. ponticum*	80 ± 3
	Uninvaded	75 ± 2
Control	Distilled Water	81 ± 2

*sativa* seedlings in the soil leachate bioassays for the four different sites. Analysis with a linear mixed effect model revealed no significantly difference (P < 0.05) between the cleared and uninvaded soil leachates. Prior to statistical analyses, the mean germination data were arcsine transformed.

## Discussion

This study found that restored vegetation communities on sites cleared of *R. ponticum* were different to that of uninvaded sites. Cleared sites had significantly higher native shrub (thus excluding *R. ponticum*) and bare ground cover, and lower grass and herbaceous species cover relative to uninvaded control sites. No difference in the cover of bryophyte species was observed between the cleared and uninvaded sites. Additionally, *R. ponticum* regeneration was also observed on all three cleared sites. Liquid chromatography-mass spectroscopy (LC-MS^n^) analysis of soil samples revealed that water-soluble, and thus bioavailable, phenolic compound concentrations in the soil were low, and therefore unlikely to affect restoration. This was supported by the *L. sativa* germination assay, where germination was not inhibited in soil leachates from cleared sites. Soil pH however was significantly lower on the invaded and cleared sites relative to the uninvaded control sites.

Our findings on native vegetation restoration corroborate those of Maclean *et al*.^31^, who found a legacy effect on understory vegetation in Scottish Atlantic oak woodlands even 30 years post *R. ponticum* clearance, with forb and grass cover significantly lower compared to surrounding uninvaded areas. Maclean *et al*.^31^ also reported that native vegetation only covered around two-thirds of cleared sites 30 years post-clearance; this also supports our findings, where higher bare ground cover was observed on sites cleared of *R. ponticum*.

Such legacy effects on vegetation communities post-clearance have been reported for several other invasive species, often occurring as a result of altered soil chemical properties^33^. This led us to hypothesise that the soil of cleared and uninvaded sites would be chemically different, in terms of phenolic compounds and pH. In turn, this would have implications for site restoration, as numerous past studies suggest certain phenolic compounds can inhibit the growth of competing species^7,17,34^.

Compounds known to be phytotoxic were identified in both litter and soil methanol extracts, as well as the litter water leachates from the uncleared site. Catechin for example is reported to enhance the invasiveness of another species, *Centaurea stoebe* Lam. in North America^18,22^. The bioactive compounds found in *R. ponticum* litter may be introduced to the soil by leaching and decomposition^17^. However, no water-soluble phenolic compounds found in *R. ponticum* were detected in any of the soils collected from the cleared sites. This was most likely due to their binding to organic matter and clay particles^35,36^, their degradation by microbes and leaching from the system by rainwater soon after their introduction^36^. Thus, although *R. ponticum* produces known phytotoxins, these may not directly influence vegetation restoration post-clearance, especially in soils that are high in organic matter, due to their capacity to bind such compounds^35,36^.

Our finding that phytotoxic compounds do not influence restoration is consistent with Del Fabbro and Prati^37^, who concluded that whilst inhibitory compounds are released by some invasive plants, they are not persistent in the soil. Whilst persistence will vary with different compounds produced by different species, most are generally rapidly adsorbed or degraded^38–40^. Grove *et al*.^41^ suggested that the allelopathic effect of some invaders on habitat restoration may vary with different timescales; in the short-term, inhibitory compounds may have a direct phytotoxic effect on native species, whilst long-term effects most likely result from altered soil microbial communities^41^. These suggestions may explain field observations made by Maclean *et al*.^42^, where no difference in native vegetation restoration was observed following the addition of activated carbon to restoring plots cleared of *R. ponticum*. Activated carbon is reported to adsorb organic compounds and thus make them unavailable to plants^43^, however if these compounds are generally not bioavailable in the field post-clearance, its addition is unlikely to have any effect in alleviating allelopathic effects on native plants. Furthermore, the use of activated carbon in studies on phytotoxicity is confounded by the fact that its addition may also alter the soil microbial community, pH and nutrient availability^43–45^. Therefore, care must be taken when interpreting results from studies using this method.

The *L. sativa* bioassay results support the suggestion that phytotoxic compounds do not influence restoration post-clearance. Germination was not inhibited in the leachate of soil collected from the cleared sites, relative to the adjacent uninvaded areas. These results are corroborated by Nilsen *et al*.^19^, who found soil organic layer leachates from beneath *R. maximum* to have no inhibitory effect on *L. sativa* germination. This is no surprise, as no water-soluble compounds were detected in soil leachates of cleared sites, as mentioned above. For a short period following their introduction into soil, the compounds may have an inhibitory effect on the germination or growth of competing species^37,41^. The compounds will then however become unavailable to plants, having no active role post-clearance^37^. This is encouraging in terms of *R. ponticum* management, as we show that once the source of these compounds is removed by clearing the canopy, they pose no barrier to native habitat restoration.

Whilst introduced inhibitory compounds may not influence site restoration, the study found mean soil pH to be 0.8 units lower on the invaded and cleared sites, relative to adjacent uninvaded control sites (Table 2). These findings agree with previous studies^6,19^ but contrast with recent findings by Maclean *et al*.^31^, who did not detect differences in soil pH post *R. ponticum* invasion in an Atlantic oak woodland. It is difficult to ascertain whether the lower pH of invaded soils was caused by *R. ponticum* invasion, or whether *R. ponticum* initially established on lower pH areas. Cross^6^ reported that *R. ponticum* was found on range of soils, varying in pH from 3.3 to 6.4. The fact that Cross^6^ found *R. ponticum* growing on calcareous soils suggests the sites included in the current study were all within its relatively large range of pH tolerance, and that soil pH did not prevent *R. ponticum* from establishing on the uninvaded control areas of the current study.

The lower soil pH could have contributed towards the higher ericaceous shrub cover of the cleared sites relative to the uninvaded control sites. This decrease in pH occurs at a critical range, as soil acidification below pH 5 increases aluminium solubility to toxic levels^46^. Aluminium toxicity limits plant growth, however shrubs such as *Calluna vulgaris* are less affected due to their relationship with ericoid mycorrhizal fungi which alleviate the effects of toxicity^46–49^. As a result, the lower soil pH following clearance could favour the shrubs found in our study, such as the commonly found *C. vulgaris*, as well as *Erica tetralix*, *Erica cinerea*, and *Vaccinium myrtillus* (Table 1S), which favour acidic soils of pH 4.5 or lower^47^. A detailed investigation of the effect of pH and aluminium toxicity was beyond the scope of the current study however, as it would require thorough testing of the varying toxicities of different aluminium species on typical heathland species.

Soil acidification post *R. ponticum* invasion therefore may create conditions favourable for ericaceous species such as *C. vulgaris* and less favourable to species such as grasses, reflecting the results of heathland restoration studies which add soil amendments to lower pH^50,51^. Heathland communities during the last century have declined throughout the UK, mainly due to land use changes^52,53^. This threatened habitat supports various bird, invertebrate and mammal species^52^. In addition, the peaty soil of heathlands is important in carbon sequestration and climate regulation^52^. The recent decline of heathlands and their global importance therefore means they are arguably of greater value than more common upland habitats such as acid grasslands; therefore, the high shrub cover observed post-clearance in this study may potentially be an unexpected benefit of *R. ponticum* invasion.

Other factors may have contributed towards the differences in the native vegetation cover of cleared and uninvaded sites. For example, it is known that the soil seedbank can become depleted following occupation by a monoculture of *R. ponticum* for longer than 20 years^30,32^. *R. ponticum* was recorded on the sites used in the current study in a 1986 survey^29^, therefore the dominant species within the seedbank post-clearance would most likely be those with the most persistent seeds. The seeds of shrubs such as *C. vulgaris*, which was common on the cleared sites (Table 1S), are known to remain viable in the soil for periods of over 50 years, allowing it to become dominant on bare ground following disturbance events such as fires^30,47^. Whilst shrub regeneration within eight years of exposing bare soil can be expected^54^, the significantly higher shrub cover on cleared sites suggests the impact of *R. ponticum* invasion on the soil has some influence on vegetation restoration post-clearance. The depleting effect of invasion on the native seedbank may also partly explain the lower percentage cover of grass and herbaceous species on the cleared sites relative to the uninvaded control sites. The findings of Maclean *et al*.^32^ corroborate this, who found the seedbank species richness of *R. ponticum* invaded and cleared Atlantic woodlands to be lower than uninvaded woodlands, with graminoids the most severely affected.

Disturbance caused by trampling during site clearance would also have been an unavoidable factor influencing vegetation restoration. Severe and long-lasting disturbance such as the cattle trampling and soil rotavation^54^ can lead to higher *C. vulgaris* cover within eight years. The short episode of trampling during *R. ponticum* clearance in the current study may have promoted *C. vulgaris* establishment, which was found on all cleared sites (Table 1S).

Although native vegetation recovery was observed, *R. ponticum* regeneration was also observed at all cleared sites. This has important implications for management, as it highlights the risk of sites reverting to *R. ponticum* dominance. *R. ponticum* recolonization appeared to occur in two ways. The most common was via young shoots re-sprouting from the cut stumps, accounting for 97% of the regeneration on the cleared sites (Fig. 3). Secondly, *R. ponticum* can re-establish from seeds, possibly as the first species to colonize the bare ground left following clearance are bryophytes, on which *R. ponticum* seeds readily germinate^6,32^. The fewer plants regenerating from seed is not surprising, due to the short-lived nature of these seeds. Maclean *et al*.^32^ found very few *R. ponticum* seeds in the seedbank of cleared sites. Our results are encouraging in relation to the control of this species, showing that by re-treating the re-sprouting stems, the main source of *R. ponticum* regeneration can be removed.

Despite *R. ponticum* regeneration from seeds being rare on the sites, it cannot be ignored. Regeneration from seed may only become apparent in the longer term, due to the slow growth of young *R. ponticum* seedlings (D. Roberts, pers. comm.) resulting in small, recently germinated seedlings remaining unseen. Additionally, the prolific seed production of each shrub means that successfully germinated seeds will accumulate over time on cleared sites provided there is a nearby seed source. Harris *et al*.^55^ suggested *R. ponticum* plant height strongly affects invasion rate, with the seeds of taller individuals travelling longer distances than those of shorter individuals. Generally, *R. ponticum* plants growing in open, exposed habitats such as heathland are shorter than those growing in woodland. As a result, the vast majority of seeds released in open habitats travel less than 10 m, with only a small proportion travelling in excess of 50 m^56^. Because of this, reinvasion of the sites used in our study from nearby seed sources would likely be slow, potentially explaining the lower numbers of *R. ponticum* plants regenerating from seed relative to cut stumps. The threat of *R. ponticum* re-establishment on cleared sites therefore appears to have two phases; during the early years post-clearance, re-sprouting stems from cut stumps are the main source of regenerating plants and will need to be re-treated with herbicide. In the longer-term, once the re-sprouting plants have died, *R. ponticum* regeneration from seed will become the main threat.

In conclusion, cleared sites were floristically different to uninvaded control sites. As hypothesised, grass and herbaceous species cover was lower on cleared sites, whilst bare ground and shrub cover was higher, consisting of typical heathland species such as *C. vulgaris* and *V. myrtillus*. However, the presence of *R. ponticum* regrowth at all cleared sites highlights the susceptibility to re-establishment, mainly due to re-sprouting from cut stumps. Our second hypothesis stated that secondary compounds introduced by *R. ponticum* would remain in the soil, having an inhibitory effect on native vegetation restoration. Water extractable concentrations of phenolic compounds from *R. ponticum* were low in soils however. This, along with the lack of an effect in the germination bioassays using soil leachates from cleared sites suggests that these compounds may become unavailable to plants and thus do not affect vegetation restoration on sites within three years of clearance, contrary to the second hypothesis. Soil pH however was found to be significantly lower on invaded and cleared sites relative to uninvaded sites. This, along with the influence of long term invasion on soil seedbanks, may contribute towards the higher shrub cover on cleared sites, due to their higher tolerance of aluminium toxicity resulting from soil acidification.

These findings could have significant implications for management. By removing the source of bioactive compounds and shading, native vegetation will recover unassisted. However, such sites are susceptible to *R. ponticum* regeneration, mainly by re-sprouting, highlighting the critical importance of continued monitoring and control for longer than the initial eight years post-clearance.

## Materials and Methods

Sampling was carried out between the 30^th^ May and 3^rd^ June 2016 on heathlands within the Nant Gwynant catchment, Snowdonia National Park (SNP), Wales. Studies on vegetation restoration are often constrained by the availability of comparable sites. In the current study we selected one uncleared site and three cleared sites at varying stages of management post *R. ponticum* clearance, which were at Sygun (53°01′02.80″N, 004°04′35.56″W), Beddgelert (53°00′45.22″N, 004°05′55.22″W), Castell (53°01′44.76″N, 004°01′35.04″W), and on National Trust land (53°00′42.55″N, 004°05′22.89″W). *R. ponticum* had been present at these sites for a period of at least 30 years prior to clearance, as they were included in a 1986 survey of *R. ponticum* invasion conducted by SNP^29^. These sites were selected from a number of other potential sites based on their similarity, in terms of environmental conditions and local land use. Each site covered relatively large areas to ensure that they were representative of typical native heathland habitats in the area.

### Site descriptions

Sites shared a similar aspect, facing the north-west, and altitude varied between 100–200 m. Sygun was a 3 ha site which remained uncleared at the time of sampling. Beddgelert (2.1 ha) was cleared in August 2013 and Castell (4.1 ha) was cleared in March 2013, both three years prior to sampling. The National Trust site (5.9 ha) was cleared during the summer of 2008, eight years prior to sampling. All sites were cleared by cutting and the material was disposed of off-site. The cut stumps were not injected with herbicide immediately post-clearance; however, sites were revisited periodically, and regrowth was treated with herbicide as part of the “monitoring” phase of management. For clarity, these sites will from now on be referred to as uncleared, 3Y-1, 3Y-2 and 8Y respectively (Table 4). Each site also had an adjacent uninvaded control site for comparison. Due to the varying lengths of time the cleared sites had regenerated for post-clearance, comparison between cleared sites was avoided.

**Table 4:** Summary of the four different sites used to study restoration post-clearance.

Site name	ID	First treatment	Date of clearance
Sygun	Uncleared	N/A	N/A
Beddgelert	3Y-1	Cutting/chipping	August 2013
Castell	3Y-2	Cutting/burning	March 2013
National Trust land	8Y	Cutting/burning	May-July 2008

At the time of sampling (May 2016), Sygun was uncleared, Beddgelert and Castell were cleared three years previously and the National Trust land cleared 8 years previously.

During the five years prior to sampling, the sites received an average 3286 mm of rain annually, and the mean air temperature was 9.1 °C (CEDA Data Repository 2016). The soils at each site were acidic podzols, the uncleared and 3Y-1 sites were of the Moretonhampstead series, whilst 3Y-2 was Laployd and 8Y was Hafren^57^. In terms of land use, all sites were subjected to low-level sheep grazing, as is typical of this habitat in upland Wales.

### Native vegetation survey

Native vegetation cover was measured at ten randomly selected sampling points within each of the uncleared and cleared sites, using a 0.5 m × 0.5 m grid quadrat. Additionally, there were ten randomly selected control sampling points for each site in adjacent areas where the vegetation resembled a native heathland habitat. To ensure the control samples had not been affected by *R. ponticum*, only areas where there were no *R. ponticum* plants or cut stumps within a 5 m radius were sampled. The percentage cover of five vegetation classes within the quadrats were recorded. Classes were defined according to functional type, including grasses (including sedges and rushes), herbs, shrubs, bryophytes and bare ground. *R. ponticum* was not included in the survey, as only native species were considered.

### *R. ponticum* regeneration post-clearance

*R. ponticum* regeneration was estimated on the cleared sites on the 27^th^ of April 2017 when deciduous vegetation was not in season, allowing for a more thorough survey of young seedlings. The number of regenerated *R. ponticum* plants within six 25 m × 25 m quadrats were counted at each site, and the mean value per hectare was calculated. Regeneration was defined either as re-sprouting from cut stumps or growth from seed, based on inspection of the plant present.

### Soil and litter chemistry

Soil samples were taken on the 30^th^ of May 2016 from the surface 10 cm at the same sampling points where vegetation cover was measured. Leaf litter samples (n = 10) were taken from the uncleared site on the 24^th^ of May, 2017; litter was not collected from the cleared sites as it was not present. Samples were stored at −20 °C then freeze-dried (LTE Scientific Lyovac, Oldham, UK) prior to analysis.

Water-soluble compounds were extracted from soil and litter samples in distilled water (2 g sample mechanically shaken in 10 mL water for 24 hours), as the focus was on the bioavailable fraction. The solutions were spun in a centrifuge (Jouan B4i, Saint-Herblain, France) at 4000 × g for 5 minutes, before the supernatant was decanted to clean tubes, and pH was measured at this stage (Fisherbrand Hydrus 500, Loughborough, UK). No phenolic compounds were identified in water leachates from the soil of any of the sites. Due to this, a methanol extraction (150 mg soil: 5 mL 70% methanol) was carried out on the litter and soil samples collected beneath the *R. ponticum* thickets of the uncleared site, to investigate whether there were any compounds present in the samples. The extracts were partially purified for LC-MS^n^ analysis using 500 mg Sep-Pak C_18_ 3 cc Vac RC cartridges (Waters Ltd, Wexford, Ireland) as “described in Hauck” *et al*.^58^. Samples were dried by rotary evaporation (Jouan RC1022, Nantes, France), then resuspended in 200 µL 70% methanol.

LC-MS^n^ analyses were conducted using a Thermo-Finnigan LC-MS system (Thermo Fisher Scientific, Waltham, USA), which consisted of a Finnigan Surveyor PDA Plus Detector, a Finnigan LTQ linear ion trap with ESI source and a 4 µm C_18_ Waters Nova-Pak column (3.9 mm × 100 mm). The autosampler tray was maintained at a constant temperature of 5 °C, whilst the column was maintained at 30 °C. The flow rate was 1 mL/min with 10% going to the mass spectrometer; 10 µL of sample was injected. The mobile phase was water/formic acid (A; 100:0.1, v/v) and methanol/formic acid (B; 100:0.1, v/v); the column was equilibrated with 95% of solvent A, with the percentage of solvent B linearly increasing to 60% over 65 minutes.

Compounds were identified using the Xcalibur version 3.0 software^59^ (Thermo Fisher Scientific, Waltham, USA) by comparing MS^2^ fragmentation data in negative or positive ion mode, with data from previous studies on *Rhododendron* spp.^60–63^, in addition to comparison with data from *R. ponticum* leaf extracts.

### *L. sativa* germination bioassays

The effect of soil leachates from the different sites on seed germination and growth was investigated in *L. sativa* (var. Buttercrunch) germination bioassays. *L. sativa* was selected as a test plant as it is an indicator species commonly used in such investigations on *Rhododendron* spp.^19,20^. Additionally, the low germination success of species commonly found on our sites such as *C. vulgaris* make them impractical for use in germination assays. For each replicate (n = 10), a Fisherbrand QL 100 filter paper (Fisher Scientific, Leicester, UK) was placed in Petri dishes (55 mm), and subsequently moistened with 2 mL of the previously described soil leachates used in chemical analyses or distilled water for the control. 20 seeds were then evenly placed on each filter paper, and mean germination percentage per plate was calculated after seven days at 20 °C, following the method of Nilsen *et al*.^19^.

### Statistical analyses

Data were analysed in linear mixed effects models fitted in the R statistical program (version 3.4.3)^64^, using the *lme4* package and the *multcomp* package for subsequent pairwise comparison. As the sites had not all been cleared at the same time, direct comparisons between them would have been inappropriate and was therefore avoided. Instead, data for cleared plots v uninvaded adjacent plots were analysed, with site included as a random effect in all models, accounting for the clustering of sampling points. Additionally, CVA along with Wilks’ lambda test was used to compare the vegetation communities. Significance was assessed at the P < 0.05 level in all tests. Prior to analyses, vegetation percentage cover data and germination percentage cover data were arcsine transformed, whilst pH data were log-transformed.

## Supplementary information

Table 1S

## Data Availability

Raw data available at DOI: 10.20391/831FED6E-0CC8-409F-BED8-2190B13F74EF.
