# Supplementary material for: Shrub establishment favoured and grass dominance reduced in acid heath grassland systems cleared of invasive Rhododendron ponticum
Source: Sci Rep. 2019 Feb 19;9:2239. doi: 10.1038/s41598-019-38573-z (PMC6381222; doi:10.1038/s41598-019-38573-z)
Supplement: Supplementary file 1 — Table 1S [file 41598_2019_38573_MOESM1_ESM.docx]

# Supplementary data – Shrub establishment favoured and grass dominance reduced in acid heath grassland systems cleared of invasive *Rhododendron ponticum*.

**Gruffydd Lloyd Jones, Max Tomlinson, Rhys Owen, John Scullion, Ana Winters, Tom Jenkins, John Ratcliffe and Dylan Gwynn-Jones**

**Table 1S**: Species list for the invaded and control sites of each of the four different sites.

| **Species** | **Uncleared** | | **3Y-1** | | **3Y-2** | | **8Y** | |
| --- | --- | --- | --- | --- | --- | --- | --- | --- |
|  | **Invaded** | **Control** | **Invaded** | **Control** | **Invaded** | **Control** | **Invaded** | **Control** |
| *Vaccinium myrtillus* | Y |  | Y |  |  |  | Y |  |
| *Thuidium tamariscinum* | Y |  |  | Y | Y |  |  |  |
| *Eurhynchium praelongum* | Y |  |  |  |  |  |  |  |
| *Pteridium aquilinum* |  | Y | Y | Y | Y |  | Y | Y |
| *Anthoxanthum oderatum* |  | Y | Y | Y |  |  |  | Y |
| *Chamerion angustifolium* |  | Y |  |  |  |  |  |  |
| *Ranunculus bulbosus* |  | Y |  | Y |  |  |  |  |
| *Rumex obtusifolius* |  | Y |  |  |  |  |  |  |
| *Cerastium fontanum* |  | Y |  |  |  |  |  | Y |
| *Holcus lanatus* |  | Y |  | Y |  |  |  | Y |
| *Rubus fruticosus* |  | Y |  | Y |  |  |  |  |
| *Potentilla erecta* |  | Y |  | Y |  | Y | Y | Y |
| *Glechoma hederacea* |  | Y |  |  |  |  |  |  |
| *Festuca ovina* |  | Y | Y | Y |  |  |  | Y |
| *Dactylis glomerata* |  | Y |  |  |  |  |  |  |
| *Molinia caerulea* |  | Y | Y |  | Y | Y | Y | Y |
| *Juncus effuses* |  | Y | Y |  | Y | Y |  | Y |
| *Hyacinthoides non-scripta* |  | Y | Y |  |  |  |  |  |
| *Rhytidiadelphus squarrosus* | | Y |  | Y |  |  |  |  |
| *Isothecium myosuroides var myosuroides* | | Y |  |  |  |  |  |  |
| *Urtica dioica* |  | Y |  |  |  |  |  |  |
| *Galium aparine* |  | Y |  | Y | Y | Y |  |  |
| *Galium palustre* |  | Y |  | Y |  |  |  |  |
| *Deschampia flexuosa* |  | Y | Y |  |  | Y | Y | Y |
| *Carex flacca* |  |  | Y | Y |  | Y | Y |  |
| *Digitalis purpurea* |  |  | Y |  |  |  |  |  |
| *Polytrichum commune* |  |  | Y | Y | Y | Y | Y | Y |
| *Calluna vulgaris* |  |  | Y |  | Y | Y | Y |  |
| *Hypnum cypressiforme* |  |  | Y |  | Y |  |  | Y |
| *Acer pseudoplatanus* |  |  |  | Y |  |  |  |  |
| *Cirsium vulgare* |  |  |  | Y |  |  |  |  |
| *Helix hedera* |  |  |  | Y |  |  |  |  |
| *Campylopus introflexus* |  |  |  | Y |  |  | Y | Y |
| *Trifolium campestre* |  |  |  | Y |  |  |  |  |
| *Eriophorum angustifolium* |  |  |  | Y |  | Y |  |  |
| *Erica cinerea* |  |  |  |  | Y | Y | Y | Y |
| *Erica tetralix* |  |  |  |  |  | Y | Y |  |
| *Sphagnum papillosum* |  |  |  |  |  | Y |  |  |
| *Sphagnum palustre* |  |  |  |  |  | Y |  |  |
| *Carex echinata* |  |  |  |  |  | Y |  |  |
| *Sorbus aucuparia* |  |  |  |  |  |  | Y |  |
| *Betula pubescens* |  |  |  |  |  |  | Y |  |
| *Galium saxatile* |  |  |  |  |  |  |  | Y |
| *Rhododendron ponticum* | Y |  | Y |  | Y |  | Y |  |
